# Exendin-4 affects calcium signalling predominantly during activation and activity of beta cell networks in acute mouse pancreas tissue slices

**DOI:** 10.3389/fendo.2023.1315520

**Published:** 2024-01-16

**Authors:** Eva Paradiž Leitgeb, Jasmina Kerčmar, Lidija Križančić Bombek, Vilijem Pohorec, Maša Skelin Klemen, Marjan Slak Rupnik, Marko Gosak, Jurij Dolenšek, Andraž Stožer

**Affiliations:** ^1^ Institute of Physiology, Faculty of Medicine, University of Maribor, Maribor, Slovenia; ^2^ Center for Physiology and Pharmacology, Medical University of Vienna, Vienna, Austria; ^3^ Alma Mater Europaea-European Center Maribor, Maribor, Slovenia; ^4^ Faculty of Natural Sciences and Mathematics, University of Maribor, Maribor, Slovenia

**Keywords:** pancreas, tissue slice, beta cell, calcium imaging, exendin-4, incretin effect, connectivity

## Abstract

Tight control of beta cell stimulus-secretion coupling is crucial for maintaining homeostasis of energy-rich nutrients. While glucose serves as a primary regulator of this process, incretins augment beta cell function, partly by enhancing cytosolic [Ca^2+^] dynamics. However, the details of how precisely they affect beta cell recruitment during activation, their active time, and functional connectivity during plateau activity, and how they influence beta cell deactivation remain to be described. Performing functional multicellular Ca^2+^ imaging in acute mouse pancreas tissue slices enabled us to systematically assess the effects of the GLP-1 receptor agonist exendin-4 (Ex-4) simultaneously in many coupled beta cells with high resolution. In otherwise substimulatory glucose, Ex-4 was able to recruit approximately a quarter of beta cells into an active state. Costimulation with Ex-4 and stimulatory glucose shortened the activation delays and accelerated beta cell activation dynamics. More specifically, active time increased faster, and the time required to reach half-maximal activation was effectively halved in the presence of Ex-4. Moreover, the active time and regularity of [Ca^2+^]_IC_ oscillations increased, especially during the first part of beta cell response. In contrast, subsequent addition of Ex-4 to already active cells did not significantly enhance beta cell activity. Network analyses further confirmed increased connectivity during activation and activity in the presence of Ex-4, with hub cell roles remaining rather stable in both control experiments and experiments with Ex-4. Interestingly, Ex-4 demonstrated a biphasic effect on deactivation, slightly prolonging beta cell activity at physiological concentrations and shortening deactivation delays at supraphysiological concentrations. In sum, costimulation by Ex-4 and glucose increases [Ca^2+^]_IC_ during beta cell activation and activity, indicating that the effect of incretins may, to an important extent, be explained by enhanced [Ca^2+^]_IC_ signals. During deactivation, previous incretin stimulation does not critically prolong cellular activity, which corroborates their low risk of hypoglycemia.

## 1 Introduction

Precise control of insulin secretion from pancreatic beta cells is crucial for maintaining homeostasis of energy-rich nutrients. The main secretagogue triggering the stimulus-secretion cascade (SSC) is glucose. Entering the cell through GLUT transporters, its metabolism increases intracellular ATP concentration, leading to the closure of ATP-sensitive K+ channels (K_ATP_), a decline in K^+^ efflux, and a subsequent increase in membrane potential. This results in the activation of voltage-dependent Ca^2+^ channels (VDCC) and entry of Ca^2+^ ions into the cell, prompting exocytosis of insulin-containing granules (1, 2).

The rise in intracellular Ca^2+^ concentration of ([Ca^2+^]_IC_) is known as the triggering signal in insulin secretion and has a central role in the SSC (3). The dynamics of [Ca^2+^]_IC_ changes is oscillatory in nature, with oscillations typically observed in two distinct time domains (4–6). After an activation delay, a transient rise in [Ca^2+^]_IC_ is followed by a stable plateau with fast, synchronized, regular oscillations with a frequency of about 5 min^-1^ and duration below 20 s (5, 7–10). This fast dynamics can be superimposed upon a slower oscillatory component with a period of several minutes (5, 9, 11, 12). As changes in [Ca^2+^]_IC_ are well synchronized with changes in membrane potential in the form of bursts or slow potentials (13–15), as well as with insulin secretion (2, 11, 16–20), they serve as a reliable proxy for evaluating beta cell function (21, 22).

While glucose is the primary stimulus driving insulin secretion, a plethora of agents, among them other nutrients, hormones, neurotransmitters, and paracrine factors, modulate beta cell function via the metabolic and neurohormonal amplifying pathways (2). Among those, glucagon-like peptide 1, a gut hormone, plays an important role in fine-tuning insulin secretion. It is secreted from enteroendocrine L-cells that are distributed along the small and large intestine, in response to meal intake (23–25). Beside glucose, di-/tri-peptides and lipids, as main nutrients stimulating L-cell secretory response, bile acids, bacterial metabolites, and prebiotics were also found to increase postprandial GLP-1 secretion (23, 25–28). Proglucagon peptide expressed in these cells is cleaved to GLP-1 by tissue specific proglucagon convertase 1/3 (28). Together with the glucose-dependent insulinotropic polypeptide (GIP), cholecystokinin and gastrin, GLP-1 is responsible for the so-called incretin effect, i.e., a greater insulin secretory response to oral glucose intake in comparison with isoglycemic intravenous glucose administration (29, 30). It is estimated that up to 70% of the insulin response to oral glucose load is due to incretin amplification (31). Loss of the incretin effect is also an important functional change in type 2 diabetes, and since beta cells seem to be resistant to even supraphysiological levels of GIP in diabetic patients (29, 32), GLP-1 receptor agonists (GLP-1RAs) have received most of the focus in pharmacological efforts to normalize postprandial glucose excursions and glycemia (33). Indeed, beside its role in glucose homeostasis, GLP-1RAs have several additional biological effects, among them reducing appetite, food intake, and delaying gastric emptying, which together leads to weight reduction, another advantageous outcome in the setting of diabesity (34). GLP-1RAs also have beneficial cardiovascular and renal effects and bear a low risk for adverse effects, including hypoglycemia. Since they have an overall favorable pharmacological profile and have recently become available in oral form, they have emerged as prominent elements in diabetes therapy, especially in high-risk patients (33, 35–37). Due to rapid degradation by DPP-4 (dipeptidyl peptidase 4), a ubiquitous enzyme, present in endothelial and epithelial cells in various tissues, prolongation of t_1/2_ has been one of the key issues in pharmaceutical development. The first GLP-1RA that circumvented DPP-4 mediated breakdown and made its way into clinical use is exenatide, a synthetic exendin-4 (Ex-4), and many others have followed (37–39).

To improve and develop new incretin-based treatment options for diabetes, it is crucial to understand the mechanisms by which GLP-1RAs potentiate insulin secretion at the cellular level. GLP-1 exerts its effects through binding to GLP-1 receptor (GLP-1R), a G-protein-coupled receptor expressed on the surface of beta cells (40). Binding of an agonist to GLP-1R leads to activation of adenylyl cyclase (AC) via the G_αs_ subunit and a subsequent increase in intracellular cAMP concentration. cAMP as a second messenger engages its two main downstream effectors, protein kinase A (PKA) and exchange protein directly activated by cAMP 2 (Epac2) (41, 42). They regulate beta cell secretion at different steps along the SSC, i.e., they seem to be able to increase the ratio of [ATP/ADP]_IC_ (43, 44), inhibit the activity of K_ATP_ channels (45–47), activate nonselective cation channels (48, 49), enhance the activity of L-type voltage-dependent Ca^2+^ channels (VDCC) (45), destabilize IP_3_ and ryanodine receptors to augment Ca^2+^ induced Ca^2+^ release (CICR) from intracellular Ca^2+^ stores (50–53) and potentiate exocytosis of insulin-containing granules (45, 54–57). It is estimated that increased Ca^2+^ influx mediates approximately 30-40% of the GLP-1RAs effect, and the rest can be ascribed to the Ca^2+^-independent potentiation of exocytosis (45, 58). Indeed, in prior research, effects of GLP-1RAs on beta cell electrical activity have been investigated into detail, but these studies focused on beta cell plateau activity (59–66). Therefore, the influence of GLP-1RAs on beta cell activation and deactivation phases, which probably play a significant role during the first phase of insulin secretion and can help assess the risk of hypoglycaemia, respectively, remain to be explored (67–69). Additionally, modulation of [Ca^2+^]_IC_ dynamics by GLP-1RAs, particularly with respect to [Ca^2+^]_IC_ oscillation properties and functional connectivity during the plateau phase, are inadequately explored in the existing literature (70).

The apparent broad range of targets that are affected by GLP-1RA activation may be biased by diverse methodological approaches used. On one hand, knowledge is pooled from different experimental models that may bear differences, ranging from human islets (44, 50, 52) and dissociated human cells (45) to isolated murine islets (48, 51, 71) and cells (46, 47), to different murine models, as well as various pancreatic beta cell lines (43, 54, 55). On the other hand, studies vary in their experimental procedures, from the protocols used in isolation of the pancreatic tissue, to the type of agonist used, and its applied concentration. For example, Ex-4, as synthetic GLP-1 analogue, has not only longer biological effects, but also stimulates a higher cAMP response (38) and produces a greater insulin response compared to equimolar doses of GLP-1 (72). Furthermore, there is a discrepancy between levels of Ex-4 used therapeutically in human medicine (73, 74) and the doses used in beta cell research that are commonly in the nanomolar range (42, 75, 76). There also are conflicting results regarding the dose-response relationships of GLP-1RAs (38, 48, 72, 77–79), and how exactly they modulate different stages of beta cell activity (48, 78, 80). In conclusion, the variability in effects of GLP-1RAs observed across different studies makes their comparison challenging.

In addition to the incretin effects at the cellular level described above, it needs to be pointed out that beta cells act collectively and in an organized manner to achieve precise regulation of blood glucose. Given the intrinsic and multifaceted heterogeneity of beta cells (81–85), intercellular communication plays a pivotal role in facilitating coordinated cellular responses to glucose and other secretagogues (8, 86, 87). While paracrine and autocrine signalling also contribute to cellular crosstalk (88–90), electrical coupling of beta cells via gap junctions consisting of connexin36 (Cx36) is recognized as the primary foundation for beta cell synchronisation by fostering the spread of depolarization and Ca^2+^ waves (8, 91). In recent years, multicellular imaging combined with network analysis has enabled quantitative assessment of collective beta cell dynamics. This approach revealed several new characteristic features of beta cell synchronized activity, such as the existence of beta cell subpopulations (92–94). Cellular connectivity can be impaired in type 2 diabetes and likely contributes to perturbation in beta cell function and insulin secretion (95, 96). As such, network metrics have emerged as powerful tools in analyzing beta cell function and have functional implications in normal and diabetic conditions (22). The effects of GLP-1RAs on beta cell connectivity and network measures are still largely unexplored. It seems that GLP-1 provides a supporting role in the preservation of beta cell glucose competence when islets are strained due to metabolic stress caused by overabundance of nutrients, inflammation, or ageing (97–101). Cellular connectivity is decreased in these conditions, and GLP-1RAs seem to be able to restore beta cell connections (42, 79). In human islets where [Ca^2+^]_IC_ oscillations are less clearly discernible than in mice, the application of GLP-1 resulted in large and synchronous deflections in [Ca^2+^]_IC,_ improving cellular coordination (79). Indeed, in a recently published detailed study of [Ca^2+^]_IC_ dynamics in human islets, Ex-4 enhanced the interconnectedness of islet subregions in islets from both healthy and diabetic donors (102). In healthy rodent islets, the effects or GLP-1RAs are less clear. While some studies confirmed increased connectivity via gap junctions during glucose stimulation (42), Hodson et al. (79) found that GLP-1 does not affect beta cell synchronicity in islets isolated from mice on a control diet, as opposed to high-fat diet-fed mice.

To better understand the impact of GLP-1RAs on beta cell intracellular calcium dynamics in normal mice during all phases of collective beta cell responses to glucose and thus provide a useful baseline for more mechanistic studies in the future, in the present descriptive study we combined the acute pancreas tissue slice method with multicellular confocal laser microscopy. Beta cell function was evaluated from both the conventional physiological and the novel network-based perspective and this approach enabled us to systematically explore activation, plateau activity, deactivation, and collective activity. Given the confirmed presence of DPP-4 in murine islets (103), in the present exploratory study we utilized the DPP-4 resistant and potent GLP-1RA, Ex-4. A range of concentrations, from picomolar to nanomolar, was used to evaluate the dose-dependency.

## 2 Materials and methods

### 2.1 Ethics statement

The study was conducted according to European and national legislation and approved by the Administration for Food Safety, Veterinary Sector and Plant Protection of the Republic of Slovenia (permit numbers U34401-35/2018-2). Experimental work was done following recommendations relating to care and work with laboratory animals to minimize animal discomfort.

### 2.2 Animals/tissue slice preparation and dye loading

Experiments were performed on twelve 8-20 weeks old male C57BL/6J mice. The mice had ad libitum access to a standard chow (Ssniff, Soest, Germany) and water and were kept in individually ventilated cages (Allentown LLC, USA) with a 12-hour day-night cycle. Acute pancreatic tissue slices were prepared as previously described (21, 86, 104, 105). In brief, mice were killed with a combination of CO2 and cervical dislocation, and the abdominal cavity was accessed via laparotomy. After the common bile duct was clamped at the major duodenal papilla, the pancreas was injected with a 1.9% low-melting point agarose (Lonza, Basel, Switzerland) at the proximal common bile duct. The agarose was dissolved in an extracellular solution (ECS) containing (in mM) 125 NaCl, 26 NaHCO3, 6 glucose, 6 lactic acid, 3 myo-inositol, 2.5 KCl, 2 Na-pyruvate, 2 CaCl2, 1.25 NaH2PO4, 1 MgCl2, 0.5 ascorbic acid and kept in a prewarmed water bath at 40°C. Immediately following the agarose injection, pancreatic tissue was cooled with ice-cold ECS, extracted from the abdominal cavity, and placed into a Petri dish containing cooled ECS. The pancreas was cut into 3-5 mm^3^ large tissue cubes, embedded into agarose and cut into 140 µm thick slices with a vibratome (VT 1000 S, Leica Microsystems, Wetzlar, Germany). The tissue slices were collected in HEPES- buffered saline (HBS, consisting of (in mM) 150 NaCl, 10 HEPES, 6 glucose, 5 KCl, 2 CaCl2, 1 MgCl2; titrated to pH=7.4 with 1 M NaOH) at room temperature. For dye loading, the slices were incubated in a solution containing 25 µg Oregon Green 488 BAPTA-1 (OGB-1, Invitrogen, Waltham, MA, USA) or 25 µg Calbryte 520 AM (AAT Bioquest, Pleasanton, CA, USA), 3.75 µl dimethylsulfoxide and 1.25 µl of 20% (w/v) Pluronic F-127 dissolved in 3.33 ml HBS at room temperature for 50 min. If not specified otherwise, all chemicals were obtained by Sigma Aldrich (St. Louis, MO, USA).

### 2.3 Stimulation protocol and calcium imaging

Before imaging, acute tissue slices were kept in substimulatory glucose concentration (6 mM) in HBS at room temperature. Individual tissue slices were transferred into the recording chamber, continuously perifused with carbonated ECS at 37°C w/or w/o glucose and GLP-1RA at concentrations as specified in protocol diagrams. Imaging was performed on Leica TCS SP5 AOBS Tandem II upright confocal system (20x HCX APO L water immersion objective, NA 1.0) and Leica TCS SP5 DMI6000 CS inverted confocal system (20x HC PL APO water/oil immersion objective, NA 0.7). Time series were acquired with a frequency of 2 Hz and resolution of 512 x 512 pixels. The calcium-sensitive dye was excited with a 488 nm argon laser and the emitted fluorescence was detected by Leica HyD hybrid detector (all from Leica Microsystems, Wetzlar, Germany) in the range of 500 - 700 nM, as previously described (10, 21, 86). Laser power was adjusted to maintain a satisfactory ratio between photobleaching and signal-to-noise ratio. Imaging plane during recording was set to approximately 15 µm below tissue slice surface to avoid imaging superficial cells that might be damaged during preparation, and optical imaging thickness was set to near 4 µm to prevent recording from multiple layers of cells.

### 2.4 Data analyses

For [Ca^+2^]_IC_ dynamics analysis, ROIs were manually selected with the help of high-resolution images and frame averaging so that the extracted time series from each ROI represented a single cell’s [Ca^2+^]_IC_ signal. Time series were exported with custom-made software (ImageFiltering, copyright Denis Špelič) and analyzed offline using in-house MATLAB (The MathWorks, Massachusetts, USA) and Python (Python Software Foundations, Delaware, USA) scripts. The effects of photobleaching were mitigated with a combination of linear and exponential fitting. Traces with major motion artefacts, distorted [Ca^2+^]_IC_ signals, and those with evident non-beta cell-like characteristics were excluded from further analysis.

To determine activation and deactivation delays in beta cells during stimulation, the beginnings and ends of [Ca^2+^]_IC_ responses were manually selected on unprocessed time series data for individual cells. For quantification of sustained oscillatory activity (e.g., plateau phase), time series were first band-pass filtered to remove noise, baseline drifts and the slow component of Ca^2+^ dynamics. The frequency band of interest was chosen by visual assessment, with 0.03-0.04 Hz and 0.2-1 Hz being used as a lower and upper cut-off, respectively. The time series with extracted fast component of Ca^2+^ oscillations were smoothed by a standard adjacent averaging algorithm and binarized, so that the values during Ca^2+^ oscillations were set to 1, and values between two Ca^^2+^^ oscillations were 0 (106). A summary of the essential steps in signal processing and the extracting of the fast oscillatory activity is provided in **Supplementary Figure 1**. The extracted binarized fast activity was used in subsequent steps of the analysis, characterizing beta cell activity in terms of frequency and duration of oscillations to calculate relative active time (AT), and inter-oscillation interval variability. The relative AT determines the proportion of time occupied by oscillations (e.g., fraction of time with elevated [Ca^2+^]_IC_ level). Inter-oscillation interval variability describes the regularity of oscillations and is defined as the ratio between the standard deviation of inter-oscillation interval length and the corresponding mean interval length.

### 2.5 Functional network analyses

We constructed functional connectivity networks to quantify collective beta cell activity, as previously described in (22). By this means, nodes represent individual cells and their locations match the cells’ physical position in tissue slice. Connections between cells were determined on the basis of the temporal similarity of [Ca^2+^] signals, denoted by correlation coefficient *R* (86, 107). The *i*-th and the *j*-th cell were considered to be functionally connected if *R_ij_* >*R*_th_, i.e., their correlation coefficient *R_ij_* exceeded a threshold value *R*_th_. A variable threshold was applied to set an average number of connections per cell to 8 (*K*_avg_=8) within each islet in the specific segment of beta cell activity. The same threshold value was than applied at different segments of beta cell activity within the same islet/recording, when analyzing specific aspect of cellular activity. Furthermore, to track the evolution of beta cell networks during the activation phase, we determined the threshold value for the first 30 min after stimulation and used this threshold value for individual subsequent 6 min intervals. In all our network analyses, changes in average node degrees *K*_avg_ were used to quantify the differences beta cell synchronicity with regards to different intervals. To gain deeper insights into the structure and heterogeneity of functional networks and to identify hub nodes, we analyzed the distributions of node degrees. This analysis examines how the number of connections per cell is distributed across the entire network. A broad and heavy-tailed distribution indicates the presence of hub nodes, which have significantly higher degrees than the majority of nodes.

### 2.6 Data presentation and statistical analyses

For statical analysis and data presentation we used GraphPad Prism 9.4.1 (GraphPad Software, San Diego, CA, USA). When data are presented as boxplots with whiskers, the boxes represent the interquartile range (IQR), which spans from the 25th and 75th percentile. The whiskers are plotted using the Tukey method, where the upper whisker extends to 1.5 times the IQR above the 75th percentile and the lower whisker extends to 1.5 times IQR below the 25th percentile. Statistical differences between groups were tested using one way analysis of variance (ANOVA) on ranks, followed by Dunn’s multiple comparison test. Asterisks denote statistically significant differences (* p < 0.05. ** p < 0.01, *** p < 0.001, ****p < 0.0001). The effect size was evaluated for all the presented data using Cohen’s *d*, and it was interpreted as small (*d* = 0.2), medium (*d* = 0.5) or large (*d* = 0.8) (108).

## 3 Results

We conducted a comprehensive investigation of the effects of Ex-4, a GLP-1RA on (i) the beta cell activity during sub-threshold glucose concentration incubation and (ii) all three phases of beta cell response to stimulatory glucose concentration: the activation, plateau phase and deactivation.

**Figure 1:**
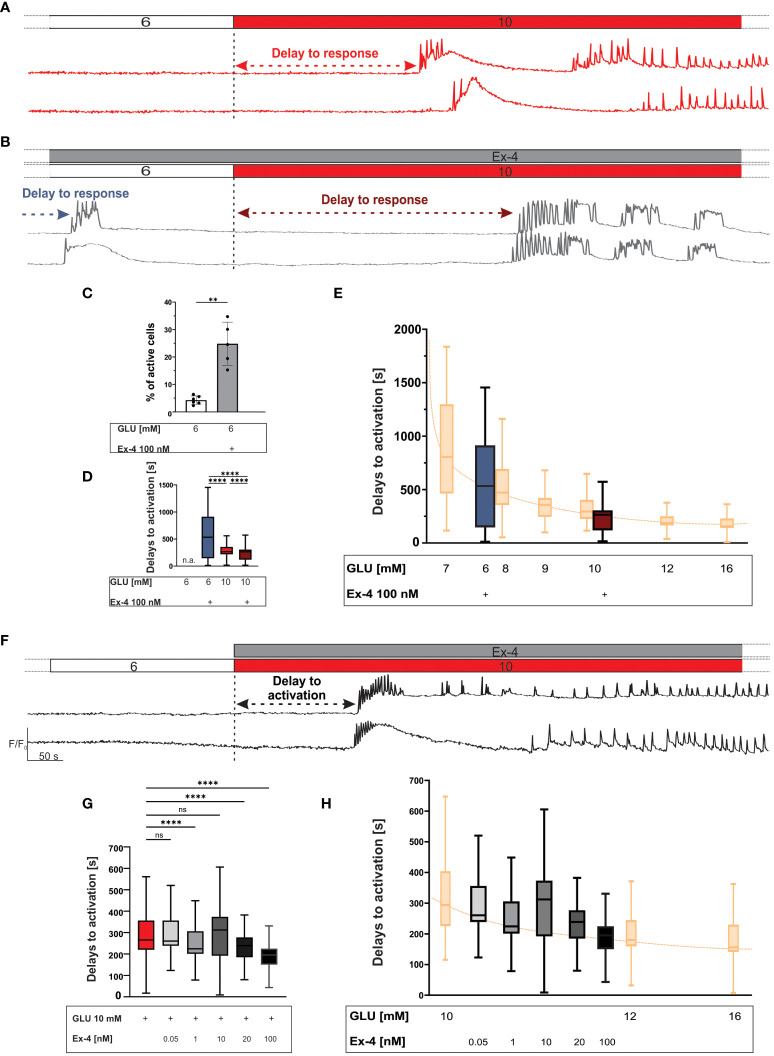
The first phase response to glucose: the effect of Ex-4 during preincubation and the effect during costimulation with glucose. **(A, B)** Protocol and representative traces of [Ca^2+^]_IC_ response to 10 mM glucose during preincubation in 100 nM Ex-4. The preincubation with Ex-4 elicited beta cell activation in sub-stimulatory glucose concentration and accelerated response to 10 mM glucose (the arrows represent the delays to activation). **(C)** Average percentage of active cells per islet in sub-stimulatory glucose (white) and with preincubation with 100 nM Ex-4 (grey), median values 4% and 25%, respectively. **(D)** Delays to beta cell activation in sub-stimulatory glucose (6 mM) and in stimulatory glucose (10 mM) with Ex-4 preincubation. Median values (in seconds) are: 533 (sub-stimulatory glucose + Ex-4 preincubation), 265 (stimulatory glucose + Ex-4 preincubation), 265 (stimulatory glucose w/o Ex-4 preincubation). To quantify the effect size, Cohen’s *d* was calculated with the following results 0.76 (6G + Ex-4 *vs* 10G), 0.92 (6G + Ex-4 *vs* 10G + Ex-4, 0.4 (10G *vs* 10G + Ex-4). **(E)** Shift of glucose-dependent activation delays due to Ex-4 preincubation (blue and dark red). Data from (109) in yellow, and sorted according to their median values. **(F)** Protocol and representative traces of [Ca^2+^]_IC_ response to 10 mM glucose during costimulation with 100 nM Ex-4. **(G)** Delays to beta cell activation in stimulatory glucose (10 mM) with Ex-4 costimulation. Median values, in seconds: 265 (10 mM glucose), 260 (0.05 nM Ex-4 + 10 mM glucose), 224 (1 nM Ex-4 + 10 mM glucose) 312 (10 nM Ex-4 + 10 mM glucose) 239 (20 nM Ex-4 + 10 mM glucose), and 195 (100 nM Ex-4 + 10 mM glucose). To assess the magnitude of the effect, Cohen’s *d* was calculated yielding following results: 0.01 (0.05 nM), 0.29 (1 nM), 0.08 (10 nM), 0.59 (20 nM), 0.65 (100 nM). **(H)** Shift of glucose-dependent activation delays due to Ex-4 costimulation. Data from (109) in yellow, and sorted according to their median values. Data are pooled from 12 different pancreas preparation, from the following number of cells/islets: 470/5 (for preincubation with Ex-4), 398/6 (for evaluation of activity in 6 mM glucose) and 6423/78 (for control response to 10 mM glucose), 278/4 (0.05 nM), 468/4 (1 nM), 1418/14 (10 nM), 257/3 (20 nM) 604/5(100 nM). The following symbols indicate p-values: *p < 0.05, **p < 0.01, ***p < 0.001, ****p < 0.0001; ns, not significant, n.a. not applicable. Effect size was interpreted as small (*d* = 0.2), medium (*d* = 0.5) or large (*d* = 0.8).

We first examined the effects of GLP-1 agonism on beta cell [Ca^2+^]_IC_ in substimulatory 6 mM glucose (**Figures 1A–D**). In the setting of our experiments, this concentration rarely induced beta cell activation (86, 109). Addition of 100 nM Ex-4 induced [Ca^2+^]_IC_ elevations, either as transient Ca^2+^ oscillations or first phase-like Ca^2+^ increases (**Figure 1B**). We quantified the number of activated cells, and while only a small proportion of beta cells exhibited a response in 6 mM glucose (4%), addition of 100 nM Ex-4 was able to activate a quarter of beta cell population (25%) (**Figure 1C**). The median activation delay was 533 seconds (**Figure 1D**).

We selected 10 mM glucose as an intermediate strength stimulus for beta cells (10, 61, 86, 109, 110) and examined the effect of preincubation with 100 nM Ex-4 (**Figure 1**). Irrespective of the preincubation, the test stimulus elicited a rise in [Ca^2+^]_IC_ in the shape of fast [Ca^2+^]_IC_ oscillations superimposed on a slower oscillatory component. The preincubation with 100 nM Ex-4 shortened the delays between the onset of glucose stimulation and the response (median value 265 seconds for glucose and glucose + Ex-4, **Figure 1D**). In an attempt to further quantify the effect of 100 nM Ex-4, we compared the delays with previously published dose-dependence data from the same animal strain (109) and ordered them according to their median values in **Figure 1E**. Although the shift could be determined only approximately, the Ex-4 elicited a right shift by about 1-2 mM glucose (**Figure 1E**), indicating a stimulatory effect equivalent or comparable to the indicated glucose increase.

Further, we tested the effect of different concentrations of Ex-4 during costimulation with the test glucose pulse (**Figures 1F–G**). Ex-4 shortened the delays in a concentration-dependent manner: median values were 265 seconds (glucose only), 260 seconds (glucose + 0,05 nM Ex-4), 224 seconds (glucose + 1 nM Ex-4), 312 seconds (glucose + 10 nM Ex-4), 238 seconds (glucose + 20 nM Ex-4) and 195 seconds (glucose + 100 nM Ex-4). In **Figure 1H** we quantified the effect of different Ex-4 concentrations using the same analysis as in **Figure 1E**, demonstrating a progressive right-shift and advancement of response, indicating Ex-4 strength of about 1-2 mM glucose at the highest concentration used.

**Figure 2:**
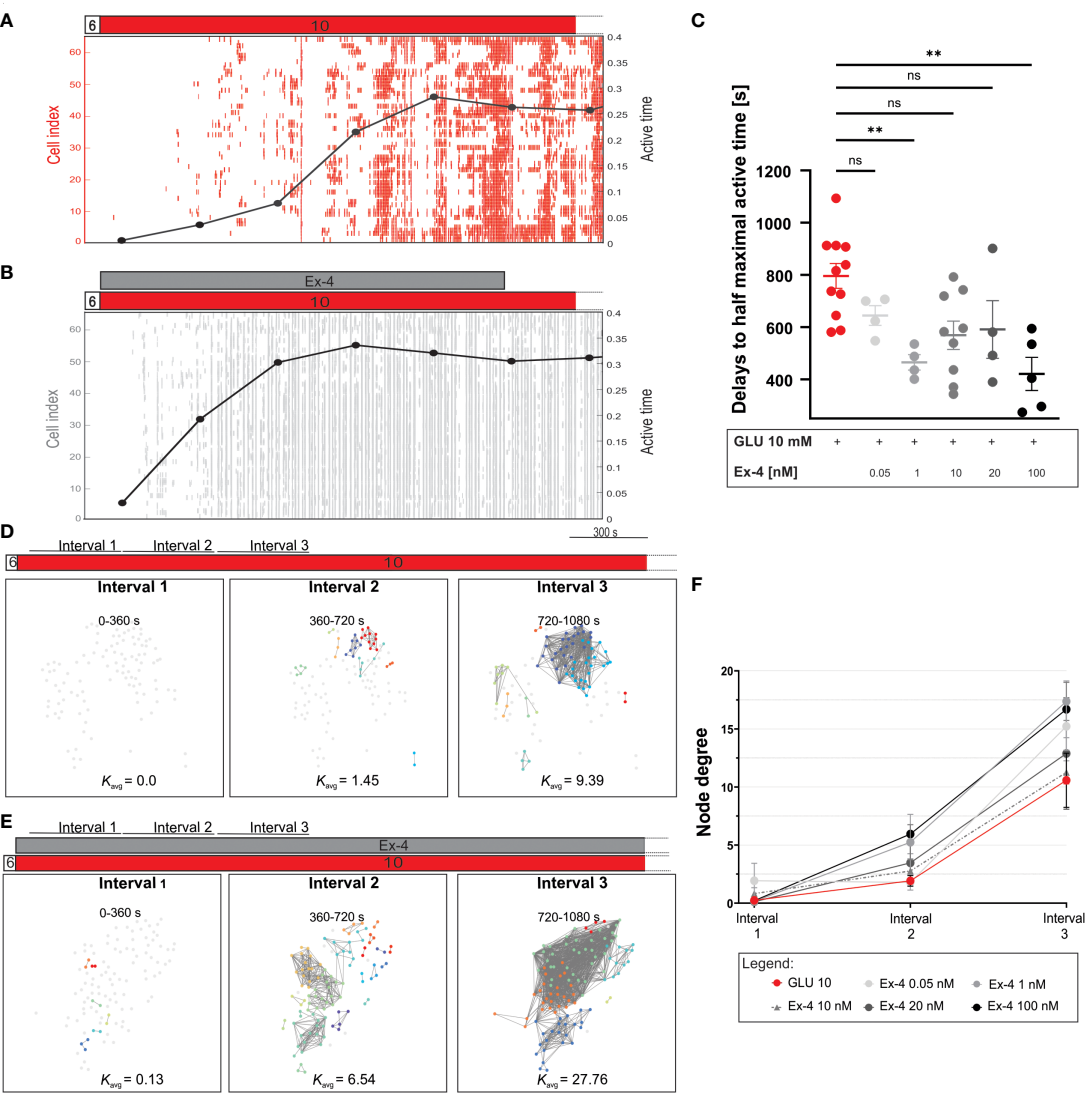
First phase of response to glucose: the effect of Ex-4 on temporal dynamics of active time and network evolution. **(A, B)** Binarized beta cell activity in representative islets during costimulation with Ex-4 and 10 mM glucose and during control conditions. Temporal evolution of the average beta cell active time is shown in black line. **(C)** Quantification of delays to half maximal active time during stimulation with different concentrations of Ex-4 and 10 mM glucose. Dots represent the time interval required for an islet to reach 50% of its maximal active time. Median values (in seconds): 816 (10 mM glucose), 662 (0.05 nM Ex-4 + 10 mM glucose), 461 (1 nM Ex-4 + 10 mM glucose), 582 (10 nM Ex-4 + 10 mM glucose), 536 (20 nM Ex-4 + 10 mM glucose), 405 (100 nM Ex-4 + 10 mM glucose) from 12 different pancreas preparation and the following number of islets 11, 4, 4, 9, 4, and 5, respectively. Cohen’s *d* values, representing the size of the effect are: 1.22 (0.05 nM), 2.77 (1 nM), 1.14 (10 nM), 1.07 (20 nM), 2.05 (100 nM). **(D, E)** Characteristic functional networks during the beta cell activation with 10 mM glucose stimulation **(D)** and when Ex-4 was added to 10 mM glucose **(E)**. To enable comparison between different islets, a variable threshold was applied to set an average number of connections per cell to 8 (*K*_avg_=8) during the first 30 minutes of beta cell activity. The same threshold value was than applied at the three intervals (intervals 1-3) encompassing beta cell activation. Each interval was 6 minutes long. **(F)** Quantification of beta cell connectivity with node degrees in the three intervals under different Ex-4 concentrations. The data are plotted as mean +/- SEM. Mean values for interval 1-3 are 0.3, 1.9, 10.6 (10 mM glucose), 1.9, 1.8, 15.2 (0.05 nM), 0.1, 5.2 17.4 (1 nM), 0.8, 2.7, 11.3 (10 nM), 0.1, 3.5, 12.9 (20 nM), 0.2, 5.9, 16.7 (100 nM), respectively. Data pooled from 12 different pancreas preparations and the following number of islets: 10 (10 mM glucose), 4 (0.05 nM), 4 (1 nM), 9 (10 nM), 4 (20 nM), 5 (100 nM). The following symbols indicate p-values: *p < 0.05, **p < 0.01, ***p < 0.001, ****p < 0.0001; ns, not significant. Effect size was interpreted as small (*d* = 0.2), medium (*d* = 0.5) or large (*d* = 0.8).

To further explore the role of incretins in beta cell activation, we investigated in detail the temporal evolution of beta cell activation phase (**Figures 2A–C**). **Figures 2A, B** illustrates binarized cellular activity during transition from inactive (before stimulation with glucose) to active state (during stimulation with glucose), demonstrating progressive increase in activity (measured as active time) to a rather steady maximal level. We noticed that the cells were recruited faster to their respective maximal activity and that, conversely, the active time under Ex-4 increased more rapidly. To quantify this effect, we analyzes the time required for beta cells to reach half-maximal active time within islets, indicating a concentration-dependent advancement of active time (**Figure 2C**). The time for half maximal activation was approximately halved at 100 nM Ex-4 (median value 816 seconds for glucose and 405 seconds for glucose + Ex-4) and the effect size was large across all tested concentrations. Complementary to active time development, we analyzed the temporal evolution of functional beta cell networks. We constructed three separate connectivity maps, each covering a 6-minute time window, starting from the onset of stimulation. The rapid activation initiated by Ex-4 described previously was confirmed again, with the density of the networks increasing already in interval 2, when control islets were still largely desynchronized (**Figures 2D, E**, middle panels). In control experiments the spatiotemporal activity was characterized predominantly by local and less coherent Ca^2+^ waves, whereas the action of GLP-1 agonist induced a higher proportion of larger and more coordinated Ca^2+^ waves that spread across wider areas of the islet (compare raster plots in **Figures 2A, B**). Increased connectivity under Ex-4 was maintained in the third temporal interval as well. To provide a more quantitative and detailed insight, we show in **Figure 2F** node degrees for different intervals and concentrations. The results indicate that for all Ex-4 concentrations the number of functional connections during subsequent time frames was higher when compared to control protocol with glucose stimulation only.

**Figure 3:**
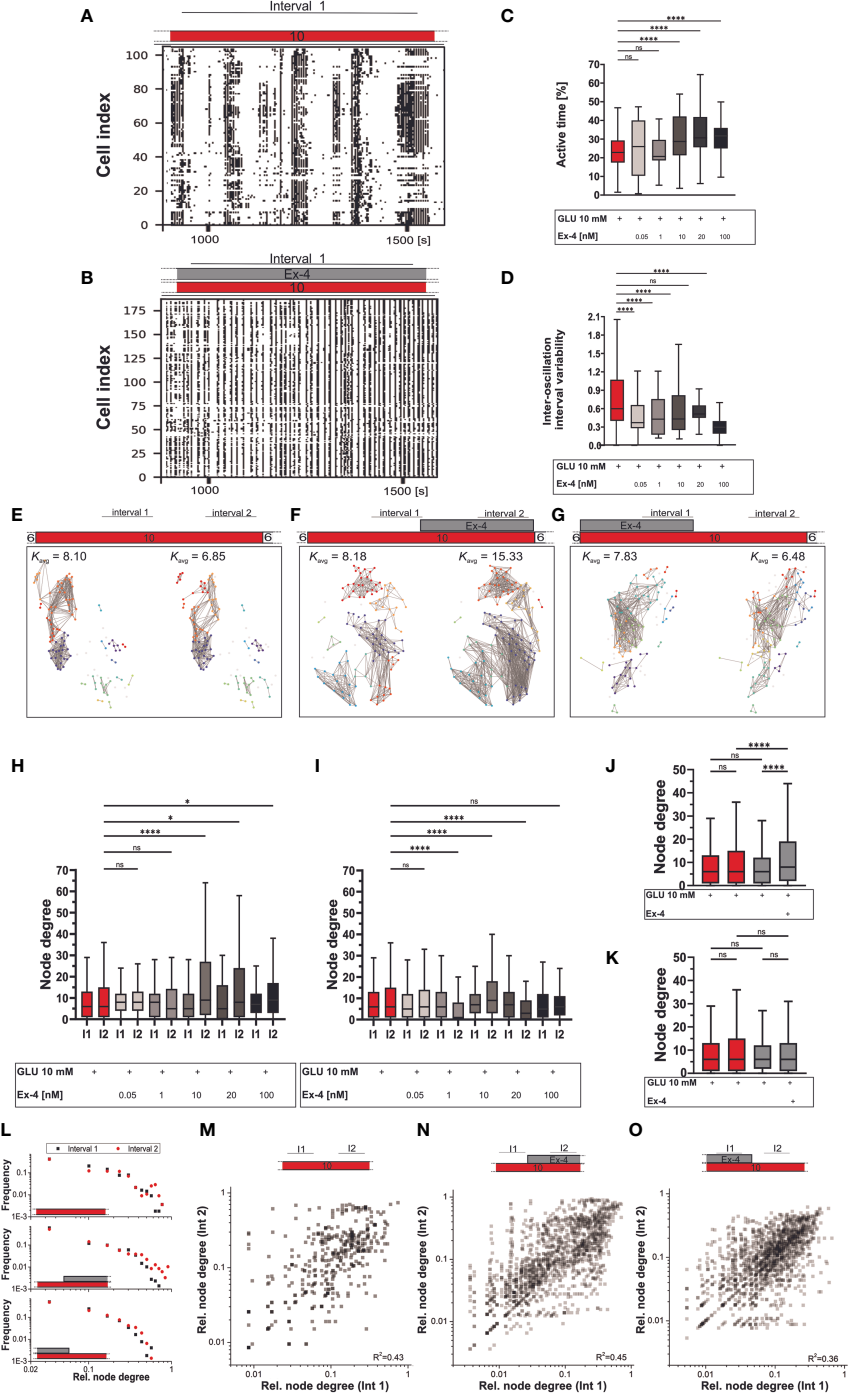
Plateau phase of glucose response: the effect of Ex-4 costimulation. **(A–D)** Binarized beta cell activity during interval 1 under glucose **(A)** and glucose with Ex-4 **(B)** stimulation in two representative islets. Interval 1 was defined as an 8–10-minute period following stabilization of cellular activity after beta cell activation. Quantification of activity during the interval 1 of the plateau phase with active time **(C)** and inter-oscillation interval variability **(D)**. Median values for active time (%): 23 (10 mM glucose), 26 (0.05 nM), 21 (1 nM), 29 (10 nM), 30 (20 nM), 32 (100 nM) and inter-oscillation interval variability: 0.60 (10 mM glucose), 0.37 (0.05 nM), 0.43 (1 nM), 0.43 (10 nM), 0.51 (20 nM), 0.30 (100 nM). The corresponding Cohen’*d* values, estimating the effect size are 0.01 (0.05 nM), 0.11 (1 nM), 0.51 (10 nM), 0.78 (20 nM), 0.66 (100 nM) for active time and 0.64 (0.05 nM), 0.71 (1 nM), 0.43 (10 nM), 0.46 (20 nM), 1.19 (100 nM) for inter-oscillation interval variability. (**E–O)** Beta cell functional connectivity. Characteristic functional networks for 10 mM glucose only **(E)**, with addition of Ex-4 in interval 2 **(F)** and interval 1 **(G)**. Nodes signify position of beta cells within an islet, connections stand for functional associations in Ca^2+^ activity. To facilitate inter-islet comparison, a variable threshold was applied to set an average number of connections per cell in an islet to 8 (*K*_avg_=8) within the first interval of beta cell activity. The same threshold value was than applied at the second interval (interval 2) of beta cell activity within the same islet. Interval 2 was defined as an 8–10-minute period occurring just before conclusion of beta cell stimulation. **(H–I)** Average node degree in interval 1 and 2 for protocol indicated in panels **(F, H)** and **(G, I)**. **(J–K)** Pooled data for all Ex-4 concentrations in protocol F **(J)** and G (**K**), where Ex-4 has been applied either in the second or in the first interval. Median values in interval 1/interval 2 for protocol under F/H: 6/6 (10 mM glucose), 8/8 (0.05 nM), 8/5 (1 nM), 5/9 (10 nM), 5/8 (20 nM), 7/9 (100 nM); for protocol under G/I: 6/6 (10 mM glucose), 5/6 (0.05 nM), 6/1 (1 nM), 7/9 (10 nM), 7/3 (20 nM), 5/6 (100 nM). For pooled data in **(J-K)** the median values for interval1/interval2 are 6/6 (10 mM glucose), 6/8 (Ex-4) **(J)** and 6/6 (Ex-4) **(K)**. To assess the magnitude of the effect, Cohen’s *d* was calculated yielding following results: 0.02 (0.05 nM), 0.17 (1 nM), 0.49 (10 nM), 0.46 (20 nM), 0.15 (100 nM) for protocol in **(H)**; and 0.12 (0.05 nM), 0.59 (1 nM), 0.23 (10 nM), 0.58 (20 nM), 0.22 (100 nM) for protocol in **(I)**. For pooled data in **(J–K)** Cohen’s *d* values are: 0.18 (int 1 (10G) *vs* int 2 (10G)), <0.001 (int 1 (10G) *vs* int 1 (Ex-4)), 0.46 (int 1 (Ex-4) *vs* int 2 (Ex-4)), 0.31 (int 2 (10G) *vs* int 2 (Ex-4)) for **(J)** and 0.18 (int 1 (10G) *vs* int 2 (10G), <0.001 (int 1 (10G) *vs* int 1 (Ex-4)), 0.11 (int 1 (Ex-4) *vs* int 2 (Ex-4)), 0.06 (int 2 (10G) *vs* int 2 (Ex-4)) for **(K)**. **(L)** Degree distribution for control data (top panel), addition of Ex-4 during interval 2 (middle panel) and interval 1 (bottom panel). **(M–O)** Relative node degree during interval 1 and 2 for individual beta cells in control experiments **(M)**, stimulation with Ex-4 in interval 2 **(N)** and interval 1 **(O)**. Individual node degrees were normalized with the network size to ensure comparison of data from different islets. The data are pooled from 12 different pancreas preparations and the following number of cells/islets: 633/10 (10 mM glucose), 390/4 (0.05 nM) 150/3 (1 nM) 737/9 (10 nM) 408/6 (20 nM) 453/5 (100 nM) (protocol in **(H)**) and 633/10 (10 mM glucose), 267/4 (0.05 nM), 288/4 (1 nM), 899/9 (10 nM), 191/3 (20 nM), 478/5 (100 nM) (protocol in **(G)**). The following symbols indicate p-values: *p < 0.05, **p < 0.01, ***p < 0.001, ****p < 0.0001; ns, not significant. Effect size was interpreted as small (*d* = 0.2), medium (*d* = 0.5) or large (*d* = 0.8).

For the plateau phase of activity, we employed two sets of experimental protocols. In the first set, beta cells were exposed to Ex-4 simultaneously with the glucose stimulation and in the second type, Ex-4 was added to perifusion after 20 minutes of initial stimulation by glucose. Concurrent initial stimulation with glucose and Ex-4 led to substantial increases in beta cell activity compared to control (**Figures 3A, B**). Results in **Figure 3C** indicate that the E-x4 increased active time in a dose-dependent manner. The active time increased up to 40%, i.e., from 23% in control experiments to 32% in 100 nM Ex-4. Notably, cellular activity also became more regular, as seen in raster plots in **Figure 3A**. This is reflected in decreases in inter-oscillation interval variability from 0.60 in 10 mM glucose to 0.30 in the highest dose of the agonist (**Figure 3D**). The effect size was medium for concentrations of Ex-4 at or above 10 nM. Regarding the effects on the inter-oscillation interval, they were of medium size even at the lowest concentration and large at the highest concentration. The addition of Ex-4 at a later stage caused an inconsistent and dose-independent increase in beta cell activity (data not shown).

To characterize beta cell collective activity during the plateau phase, the stable part of beta cell activity was divided into two intervals, as indicated in protocols in **Figure 3**. Functional networks were constructed for each interval. Panels E-G in **Figure 3** show characteristic beta cell networks under various stimulation protocols with Ex-4 (**Figures 3F, G**) and control conditions (**Figure 3E**). To establish a consistent baseline, we employed an adaptive threshold approach and set the average degree in the first interval of the plateau phase to *K*_avg_= 8. Prolonged exposure to glucose alone maintained the connectivity among beta cells throughout the recording, thus preserving the network density in interval 2 (**Figure 3E**, red data in **Figures 3H–K**). Addition of Ex-4 in the second half of stimulation (**Figure 3F**) produced an increase in connectivity, i.e., in average node degree. Conversely, when Ex-4 was administered at the beginning of the stimulation (**Figure 3G**), cellular connectivity throughout the course of stimulation remained nearly unaltered. Correspondingly, **Figure 3H**, which represents data from the protocols where Ex-4 was applied in the second half of stimulation, shows increase in number of functional connections for ≥ 10 nM Ex-4 compared to glucose only stimulation. In contrast, if Ex-4 was applied in the first half of stimulation with glucose (**Figure 3G**), the average degrees did not display a clear Ex-4 dose-dependence and in this case the network structures remain unaltered (**Figure 3I**). Pooling the data from all protocols with Ex-4 stimulation in the second interval (**Figure 3J**) and initial costimulation with Ex-4 (**Figure 3K**) confirmed this. The impact of Ex-4 on beta cell functional connectivity ranged from small to medium in terms of effect size.

As indicated by their heterogenous degree distribution, beta cell networks, under all experimental conditions and both intervals, exhibited the characteristics of broad-scale networks (**Figure 3L**). The heavy-tailed nature of the degree distribution obtained in all cases indicates the presence of hub cells. Notably, during control experiments and experiments involving Ex-4 application in the second interval there was a slight increase in the heterogeneity of degree distribution, whereas it remained unaltered during experiments with initial costimulation with Ex-4. Furthermore, the role of individual beta cells in the functional network, as determined by their degrees, remained consistently stable, irrespective of the specific protocol they were exposed to (**Figures 3M–O**).

**Figure 4:**
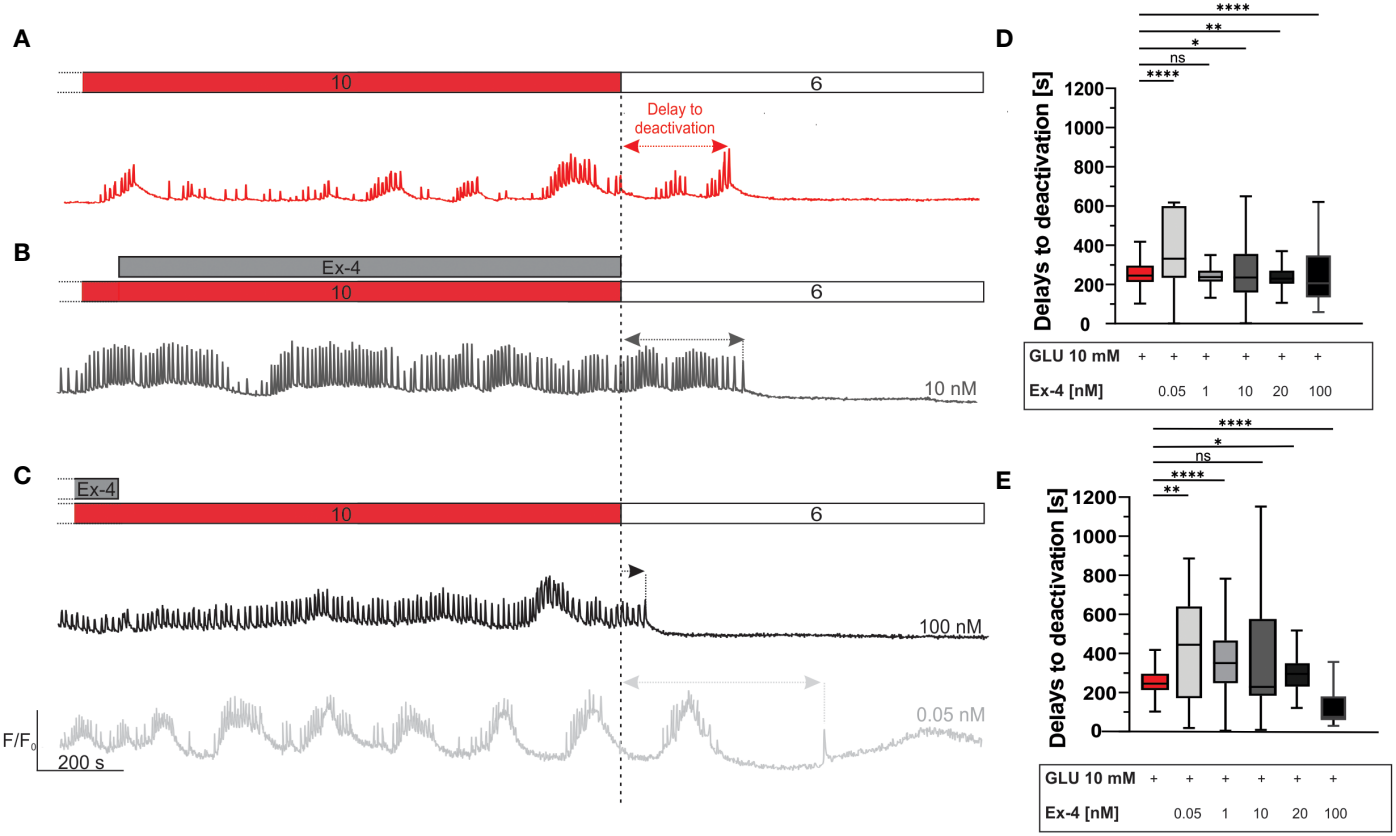
Beta cell deactivation: the effect of Ex-4. **(A–C)** Schematic representation of delays to deactivation after cessation of stimulation. The delays to deactivation are measured as the time difference between end of stimulation (dashed line) and the last [Ca^2+^]_IC_ oscillation. Shown are representative traces from three different stimulation protocols - glucose stimulation only (red) **(A)**, application of 10 nM Ex-4 (grey) during the second interval **(B)** and application of 100 nM (black) and 0.05 nM (light grey) Ex-4 during first interval of beta cell stimulation **(C)**. Pooled data are shown in **(D, E)**. **(D)** Delays to deactivation for glucose and different concentrations of Ex-4 in protocol **(B)**. Median values, in seconds: 245 (10 mM glucose), 331 (0.05 nM), 238 (1 nM), 236 (10 nM), 229 (20 nM), 206 (100 nM). **(E)** Delays to deactivation for glucose and different concentrations of Ex-4 in protocol **(C)**. Median values, in seconds: 245 (10 mM glucose), 444 (0.05 nM), 350 (1 nM), 229 (10 nM), 295 (20 nM), 74 (100 nM). To assess the size of the effect, Cohen’s *d* was calculated yielding following results: 0.45 (0.05 nM), 0.38 (1 nM), 0.11 (10 nM), 0.32 (20 nM), 0.13 (100 nM) for protocol in **(D)**; and 0.66 (0.05 nM), 0.52 (1 nM), 0.40 (10 nM), 0.02 (20 nM), 1.04 (100 nM) for protocol in **(E)**.Data are pooled from 12 different pancreas preparations and from the following number of cells/islets: 826/11 (10 mM glucose), 363/3 (0.05 nM), 178/3 (1 nM), 767/8 (10 nM), 386/7 (20 nM) 478/6 (100 nM) for data in **(D)** and 277/4 (0.05 nM), 359/3 (1 nM), 998/9 (10 nM), 302/4 (20 nM), 535/5 (100 nM), for data in **(E)**. The following symbols indicate p-values: *p < 0.05, **p < 0.01, ***p < 0.001, ****p < 0.0001; ns, not significant. Effect size was interpreted as small (*d* = 0.2), medium (*d* = 0.5) or large (*d* = 0.8).

Finally, we investigated the pattern of beta cell deactivation after removal of stimulus, to assess the possibility of prolonged deactivation delays that could correlate with continued insulin secretion and risk for hypoglycemia in type 2 diabetics (67, 111). Following the two protocols described above (i.e., Ex-4 costimulation during the first half or the second half of the 40-minute glucose stimulation interval), perifusion was switched back to the sub-stimulatory concentration of 6 mM. Following a delay, this led to beta cell deactivation, as illustrated in **Figures 4A–C**. Median deactivation delay was 245 s for control experiments. Experiments involving Ex-4 deactivation delays exhibited a U-shaped dose-dependence. Specifically, lower concentration of Ex-4 during the second half of glucose stimulatory interval prolonged the deactivation delays (median value 331 seconds in 0.05 nM Ex-4) and higher concentration expedited beta cell deactivation (median value 206 seconds in 100 nM Ex-4) (**Figures 4B, D**). This phenomenon was even more pronounced during the costimulation in the first half of glucose stimulation interval (**Figures 4C, E**): lower concentrations of Ex-4 resulted in a notable extension of beta cell activity (median delay 444 seconds in 0.05 nM and 350 s in 1 nM Ex-4), and 100 nM shortened deactivation delays to a median value of 75 seconds (**Figure 4E**). The effect size was the largest at both ends of the concentration spectrum in experiments with initial costimulation with Ex-4. In the low concentration range, the effect size was medium to large, and in the highest concentration it was large.

## 4 Discussion

To gain a better insight into how GLP-1R agonism influences beta cell calcium dynamics, we here systematically analyzed classical physiological parameters characterizing beta cell activation, plateau phase activity, and deactivation, as well as beta cell collective behavior by extracting and quantifying functional beta cell networks.

As literature reports diverse effects in regard to GLP-1R activation (38, 42, 48, 72, 77–80, 112), we opted to assess a range of concentrations to see if this heterogeneity might, in part, be attributed to variations in the concentrations utilized. The therapeutic concentration of Ex-4 is in the picomolar range and it closely approximates the lowest concentration we tested. However, in beta cell research nanomolar concentrations are often utilized (42, 72, 76, 113), and were therefore included in the range we tested. We did not study additional GLP-1RA, such as GLP-1 or liraglutide used in other studies, due to the large number of different protocols and concentrations in the present study, but testing at least some of other agonists at selected concentrations and in selected protocols remains a realistic goal for future that would enable head-to-head comparisons and insights into possible therapeutically relevant differences.

We started with investigating the effect of Ex-4 in 6 mM glucose. This concentration of glucose is close to fasting levels in mice (114, 115) and, in the context of our experiments, sits just beneath the activation threshold (10, 53, 86, 109). In the present work, this was confirmed again with only 4% of beta cells showing activity at this substimulatory glucose level. In contrast, Ex-4 was able to instigate [Ca^2+^]_IC_ elevations qualitatively resembling activity at glucose concentrations higher by approximately 2 mM in a quarter of beta cells (**Figure 1C**). The delays to activation in 6 mM glucose with Ex-4 were long (533 seconds) (**Figures 1D, E**) and were comparable to delays found in 7-8 mM glucose, as established in previously published data from the same mouse strain and methodological approach (109). Our findings are also in good agreement with previously published studies demonstrating that while the GLP-1 signalling pathway is ineffective when glucose is low or absent (38, 43, 52, 66, 116), subthreshold glucose levels are sufficient for inducing membrane depolarization (52, 60, 62, 65, 66, 110, 117) and [Ca^2+^]_IC_ increases (43, 48, 52, 66, 70, 116–118) in at least a part of islet beta cells and that this activity resembles activity at higher glucose levels. The most pronounced effects have typically been observed with direct activation of adenylate cyclase by forskolin, however the effects in our study are more similar to previously described effects of GLP-1RAs, synthetic cAMP analogues, and phosphodiesterase inhibitors that exhibited comparatively weaker effects (60, 62, 110). As hypoglycaemia is one of the main considerations in evaluating the safety of pharmaceuticals which potentiate insulin secretion, and GLP-1RA are considered low risk for hypoglycaemia due to their glucose-dependent insulinotropic action (119), activation of GLP-1R in substimulatory glucose seems contradictory. However, given that in our experiments even the most potent concentration of Ex-4 in 6 mM glucose could only elicit a [Ca^2+^]_IC_ response in approximately a quarter of beta cells within an islet, and that these [Ca^2+^]_IC_ elevations were transient in nature (**Figure 1B**), it is improbable that this could contribute to a substantial insulin secretion capable of triggering a potentially life-threatening hypoglycaemia. Furthermore, as discussed under deactivation below, *in vivo* even a small increase in insulin secretion would lower the baseline glucose level and probably terminate the response to Ex-4.

For stimulatory conditions, we increased glucose to 10 mM, which is an intermediate stimulatory concentration sufficient for activating most beta cells, but as it approximates non-fasting levels in mice (109), it is still low enough to be considered physiological. Importantly, at 10 mM glucose beta cell function is not saturated and this concentration is better suited to discern possible potentiating effects of Ex-4 compared with higher supraphysiological concentrations of glucose (10, 62, 109, 120). Previous research has shown that beta cell activation delays are progressively shorter with increasing glucose concentrations (10, 86, 109). In our experiments, preincubation with Ex-4 did not significantly decrease the median delay to activation in 10 mM glucose (**Figures 1D, E**), but initiated a rapid response to high glucose in a subpopulation of beta cells. Costimulation with Ex-4 had a considerably more potent effect and shortened the activation delays in a concentration-dependent manner (**Figure 1D**), with the highest Ex-4 concentration approaching values in 12 mM glucose (**Figure 1H**), again indicating increased sensitivity corresponding to a 1-2 mM increase in glucose. Similar effects were previously observed in beta cell electrical activity induced by increasing beta cell cAMP levels with theophylline or forskolin (62, 110).

Our findings further demonstrate that in presence of Ex-4 beta cells reach their half-maximal active time significantly earlier, with the highest dose of Ex-4 halving this time compared to controls (**Figure 2C**). The evolution of functional beta cell networks further supports this finding, revealing increased beta cell connectivity with higher number of functional connections during beta cell activation under Ex-4, compared with control islets that were during activation weakly synchronized, as indicated by sparser networks, predominantly local [Ca^2+^]_IC_ waves, and a lower average node degree (**Figure 2**). These results suggest that GLP-1RAs not only accelerate beta cell recruitment but also lead to a high level of coordination in the responses. Since the temporal evolution of [Ca^2+^]_IC_ during the activation phase in **Figure 2** closely corresponds to the first phase of biphasic insulin secretion (16, 69), our findings strongly suggest increased insulin secretion during this phase, but this needs to be further assessed in the future.

Beta cell activation is followed by the stable plateau phase. This is the most extensively studied aspect of beta cell activity, characterized by repetitive changes in membrane potential, correlating with fast [Ca^2+^]_IC_ oscillations and pulsatile insulin secretion (4, 13, 121). In the present paper, costimulation with glucose and Ex-4 dose-dependably increased active time during the initial part of stable cellular activity (interval 1) by up to 40% (**Figure 3C**). This increase was primarily mediated either through an increase in oscillation frequency (10 nM Ex-4) or both duration and frequency (20 and 100 nM Ex-4). In all instances, the primary factor contributing to the observed changes in active time was the increase in frequency. Specifically, the frequency rose from 0.056 Hz in 10 mM glucose to 0.069 Hz (20 nM Ex-4) and 0.074 Hz (10 and 100 nM Ex-4), representing a 20-32% increase. In contrast, the prolongation of the oscillations amounted to only around 10¸%, i.e., from 3.8 seconds in glucose only to 4.1 seconds with additional 100 nM Ex-4 (**Supplementary Figure 2**). Qualitatively similar increases in beta cell active time have also been observed in previous studies investigating beta electrical activity and [Ca^2+^]_IC_ dynamics (60–62, 65, 122). However, how exactly beta cells achieve a greater active time seems to differ between various agonists. GLP-1, cAMP analogues, low doses of PDE inhibitors, and even forskolin in one study mainly prolong the burst of membrane depolarization (59, 63, 65, 110, 122), while the shortening of intervals between bursts is not as prominent, leading only to a slight increase in frequency of the burst (59, 62, 63). High doses of theophylline on the other hand decreased both the length of the burst and intervals between them, increasing fraction of the plateau phase only through the increased frequency (62). A recent study of a different GLP-1RA, liraglutide, also showed an increase in [Ca^2+^]_IC_ oscillatory frequency (123), as did most experiments with forskolin, where the beta cell active time was elevated by a prominent increase in the frequency of [Ca^2+^]_IC_ oscillations (61, 70), even when their duration decreased (70). Quantitatively, the effect on beta cell active time in previous studies is also comparable to our results, but is heterogeneous and ranges from 24-100% with GLP-1 (79, 124), 30-80% with different doses of theophylline (62), 30-100% with forskolin (61, 70, 110) to approximately 40% with Ex-4 (102), and 100% with dbcAMP (62, 110) on the background of comparable glucose levels (9-12 mM).

Our current study is principally descriptive in nature and was intended to systematically screen for and quantitatively characterize the effects during the different phases of beta cell activity and the mechanisms behind these effects remain to be investigated into detail in future studies. However, among the different possible mechanisms promoting beta cell function during activation and activity in the presence of GLP-1RAs, a plausible contribution to the enhanced [Ca^2+^]_IC_ dynamics observed in the present study likely stems from potentiating effects on L-type voltage-dependent Ca^2+^ channels (45, 52, 116, 124), together with activation of Ca^2+^ induced Ca^2+^ release from intracellular stores (51, 52, 125–127). However, evidence also exists that activation of hyperpolarization-activated cyclic nucleotide-gated channels by GLP-1RAs plays a role in augmenting beta cell [Ca^2+^] oscillations (2, 123). Additionally, cAMP mediated inhibition in Na^+^/K^+^ ATPase activity could support beta cell depolarization, joining efforts with K_ATP_ channels (128). Furthermore, in addition to closing K_ATP_ channels, GLP-1 augments an inward current through TRPM2, TRPM4, and TRPM5 (48, 66). This enhancement could partially elucidate the faster depolarization and shorter activation time observed in cells stimulated with Ex-4. Notably, TRPM2 depletion has been shown to reduce the capacity of glucose to induce insulin secretion and elevate [Ca^2+^]_IC_ (48). The burst duration is believed to be determined by the activity of calcium-sensitive potassium channels (129) or the slower of the two inactivation components of VACCs (130, 131) or both. Given the increase in burst duration in our study, in addition to other possible effects, GLP-1 agonism could act to decrease the sensitivity of the calcium-sensitive channels to calcium, slow down the slower inactivation component of the VACCs, or both. The idea that K_ATP_ channel closure alone is insufficient to explain the stimulatory effect of GLP-1 is supported by the observation that the removal of extracellular Na^+^ abolishes the electrical activity triggered by GLP-1. Additionally, the knockout of TRPM4 has been demonstrated to prevent GLP-1-mediated depolarization and stimulation of insulin secretion (66).

Moreover, the gain in active time in our experiments corresponded with a decrease in inter-oscillation interval variability, indicating that beta cells were not only more active, but their oscillations also became more regular (**Figure 3D**). Increased regularity has been observed also with other factors potentiating beta cell function (106) and, specifically, is also in agreement with the effect of cAMP on beta cell electrical activity. Beside the increase in beta cell electrical activity, forskolin and GLP-1 also stabilized the duration of bursts and intervals between them, leading to a very regular electrical activity (63, 124). Under normal conditions, cAMP levels oscillate in beta cells with a period similar to slow cyclic variations in membrane potential and [Ca^2+^]_IC_ and is phase with them (132). Since GLP-1R signalling can change the dynamics of cAMP oscillations (132–134) changes in dynamics of cAMP could contribute to the increased stability of [Ca^2+^]_IC_ oscillations via their effects on the abovementioned targets. Additionally, increased coupling has been shown to stabilize the bursting behavior in beta cells and this is another possible explanation for the increased stability of intervals between fast oscillations. Interestingly, increased coupling could also account for a part of the increase in duration of fast oscillations (135–137), but the exact mechanisms leading to increased regularity of oscillations remain to be explored in the future.

Further, construction and quantification of functional beta cell networks has emerged as an important tool for investigating complex spatiotemporal [Ca^2+]^_IC_ dynamics. It has enabled us an insight into beta cell collective activity in healthy and diabetic islets and has become a prominent tool for the assessment of pharmacological effects on beta cell function (22, 79, 85). Several studies have confirmed the capability of GLP-1RAs to restore beta cell functional connectivity following its decreases upon exposure to high-fat diet (79) or cytokines (42), and increased GLP-1 levels have also been found to contribute to enhanced correlation in beta cell [Ca^2+^]_IC_ responses following bariatric surgery in mice (138). However, while it seems that a normal or sufficient level of cAMP, secured via GLP-1R activation (101), is needed to enable basic beta cell coupling (139), the effects of stimulated GLP-1R signalling on healthy beta cell collectives remain less clear. Farnsworth et al. observed that gap junction coupling in healthy mouse islets exposed to Ex-4 is increased, but this effect was not observed in human islets (42). Contrary to this, Hodson et al. found that in human islets, which tend to exhibit more stochastic [Ca^2+^]_IC_ responses, GLP-1R activation revealed a subpopulation of beta cell responding to GLP-1 with additional large and synchronous rises in [Ca^2+^]_IC_ (79). However, they did not see any effect of GLP-1 in healthy mouse islet. We found that Ex-4 increases beta cell collective activity when added in the second part of plateau phase, as indicated by a rise in connectivity during interval 2 in **Figures 3H, J**. Initial costimulation, on other hand, preserved the number of beta cell connections from interval 1 to interval 2, similarly to control experiments without Ex-4 (**Figures 3I, K**). It is important to note that this is likely related to our methodological approach. More specifically, we used a variable threshold, such that the average node degree in interval 1 was always set to 8 for different protocols. The same threshold was subsequently applied during interval 2 within the same islet, allowing for a direct comparison of interval 2 relative to interval 1 while pooling data from different islets. The preservation of network density during interval 2, despite initial costimulation with Ex-4 in interval 1, can be attributed to a higher threshold value established in interval 1, given the higher overall synchronicity observed in that interval. Subsequently, during interval 2, when Ex-4 was no longer present, node degree remained constant or was even lower than in interval 1. Therefore, the relative density of networks did not change from interval 1 to 2 in this protocol, although Ex-4 increased the network density *per se* (see **Figure 2**). Nevertheless, the enhanced connectivity under GLP-1RA can be linked either to the increased regularity of oscillations (**Figure 3D**), to a rise in intercellular connectivity, or to a combination of both factors. In addition to possible mechanisms accounting for the increased regularity of fast oscillations briefly described in the previous paragraph, cAMP can modify cell-to-cell communication among beta cells through different mechanisms, such as altering Cx36 gene expression, enhancing Cx36 coupling, or modifying the distribution of Cx36 on the cell membrane (41, 95). Which of these factors is more decisive for a higher level of coordination of intercellular signals remains to be explored in future studies. Finally, we noted that the functional beta cell networks in different protocols and intervals had some common characteristics. Specifically, the distribution of functional connections was heterogenous in all the networks we constructed in the plateau phase, supporting the existence of highly connected hub cells in all of the tested protocols (**Figure 3L**). Moreover, their role remained stable throughout the plateau phase (**Figures 3M–O**).

Lastly, we examined the impact of Ex-4 on beta cell deactivation properties after removal of the stimulus. This aspect of beta cell function is the least explored part of beta cell function and is typically overlooked in beta cell research. To the best of our knowledge, only one other study looked into deactivation proprieties in similar conditions, and found deactivation was prolonged by 22% after beta cells were stimulated with forskolin, a much more potent adenylyl cyclase activator (70), an effect which is quantitatively similar to supraphysiological glucose concentrations (10, 109). Our results are compelling, as concentrations on the different ends of the spectrum had opposing results. The lowest, picomolar concentration of Ex-4 slightly prolonged the delays to deactivation, while the highest, i.e., 100 nM, dose led to an earlier beta cell deactivation (**Figure 4**). As prolonged deactivation could pose a risk for episodes of hypoglycaemia in patients treated with GLP-1RAs (67, 111), it is worth emphasizing that even the longest median delays to deactivation were below 10 minutes and that beta cell oscillatory activity ultimately did cease in all cases. Additionally, *in vivo*, a slightly increased insulin secretion during deactivation would be expected to lead to a slight further decrease in glucose concentration and accelerate beta cell deactivation before hypoglycemia would ensue. The mechanism behind the accelerated deactivation at the highest Ex-4 concentration remains to be investigated, but it could reside in a further increased intercellular coupling compared to lower concentrations, which would be expected to propagate hyperpolarizing deactivating influences from cells that deactivate among the first more efficiently (67).

In sum, our results based on [Ca^2+^]_IC_ dynamics indicate that under normal, i.e., nondiabetic conditions, GLP-1 agonism mainly supports beta cell activation and increase in activity during the initial part of the response to glucose. From a homeostatic point of view, it seems that when it comes to the triggering [Ca^2+^]_IC_ signal, GLP-1R pathway enhances the role of the beta cell as a differential or phasic controller role and to a lesser extent their role as difference or tonic controllers (10, 140). This is further compatible with the view that under normal conditions this pathway primarily serves to prime beta cells and prevent dramatic postprandial increases in glucose (140). Future studies need to resolve whether different intracellular targets mediate the influences on activity during different phases and whether these are differently susceptible to diabetogenic insults.

## 5 Conclusions

Ex-4 was able to weakly activate approximately a quarter of beta cells exposed to substimulatory glucose. Initial costimulation with Ex-4 and stimulatory glucose led to a reduction of the activation delays and an acceleration of beta cell activation dynamics. In the presence of Ex-4 the active time increased faster, and time needed to reach half-maximal activation was halved. Even more, beta cell activity was greater and more regular during the initial part of the response with costimulation. However, when Ex-4 was added to already active beta cells, it did not induce as robust an increase in activity. Network analysis provided additional validation, demonstrating increased connectivity during activation and activity, with cell roles remaining stable both in control experiments and experiments with Ex-4. Of particular interest, Ex-4 slightly prolonged beta cell deactivation at the lowest concentrations and led to shorter deactivation delays in the highest concentration. In summary, costimulation by Ex-4 and glucose enhances [Ca^2+^]_IC_ during beta cell activation and activity. This suggest, the increased beta cell function under incretin stimulation may, to a significant effect, be attributed to increased [Ca^2+^]_IC_ signals.

## Data availability statement

The raw data supporting the conclusions of this article will be made available by the authors, without undue reservation.

## Ethics statement

The animal study was approved by Administration for Food Safety, Veterinary Sector and Plant Protection of the Republic of Slovenia (permit numbers U34401-35/2018-2). The study was conducted in accordance with the local legislation and institutional requirements.

## Author contributions

EP: Conceptualization, Data curation, Formal analysis, Investigation, Methodology, Project administration, Visualization, Writing – original draft, Writing – review & editing. JK: Data curation, Investigation, Writing – review & editing. LB: Data curation, Investigation, Writing – review & editing. VP: Data curation, Investigation, Writing – review & editing. MK: Data curation, Investigation, Writing – review & editing. MR: Conceptualization, Funding acquisition, Resources, Writing – review & editing. MG: Conceptualization, Formal analysis, Software, Supervision, Visualization, Writing – original draft, Writing – review & editing. JD: Conceptualization, Data curation, Formal analysis, Investigation, Methodology, Software, Supervision, Validation, Visualization, Writing – original draft, Writing – review & editing. AS: Conceptualization, Formal analysis, Funding acquisition, Methodology, Project administration, Resources, Supervision, Validation, Writing – original draft, Writing – review & editing.
