# Supplementary material for: Exendin-4 affects calcium signalling predominantly during activation and activity of beta cell networks in acute mouse pancreas tissue slices
Source: Front Endocrinol (Lausanne). 2024 Jan 16;14:1315520. doi: 10.3389/fendo.2023.1315520 (PMC10826511; doi:10.3389/fendo.2023.1315520)
Supplement: Supplementary file 1 [file DataSheet_1.docx]

***Supplementary Material***

**Exendin 4 affects calcium signalling predominantly during activation and activity of beta cell networks in acute mouse pancreas tissue slices: Implications for incretin-induced amplification of beta cell function**

**Eva Paradiž Leitgeb^1^, Jasmina Kerčmar^1^, Lidija Križančić Bombek^1^ Vilijem Pohorec^1^, Maša Skelin Klemen^1^, Marjan Slak Rupnik^1,2,3^, Marko Gosak^1,4^, Jurij Dolenšek^1,4^, Andraž Stožer^1^**^, *^

^1^ Institute of Physiology, Faculty of Medicine, University of Maribor, Maribor, Slovenia

^2^Center for Physiology and Pharmacology, Medical University of Vienna, Vienna, Austria

^3^Alma Mater Europaea-European Center Maribor, Maribor, Slovenia

^4^Faculty of Natural Sciences and Mathematics, University of Maribor, Maribor, Slovenia

***Correspondence:**   
Andraž Stožer  
[andraz.stozer@um.si](mailto:andraz.stozer@um.si)


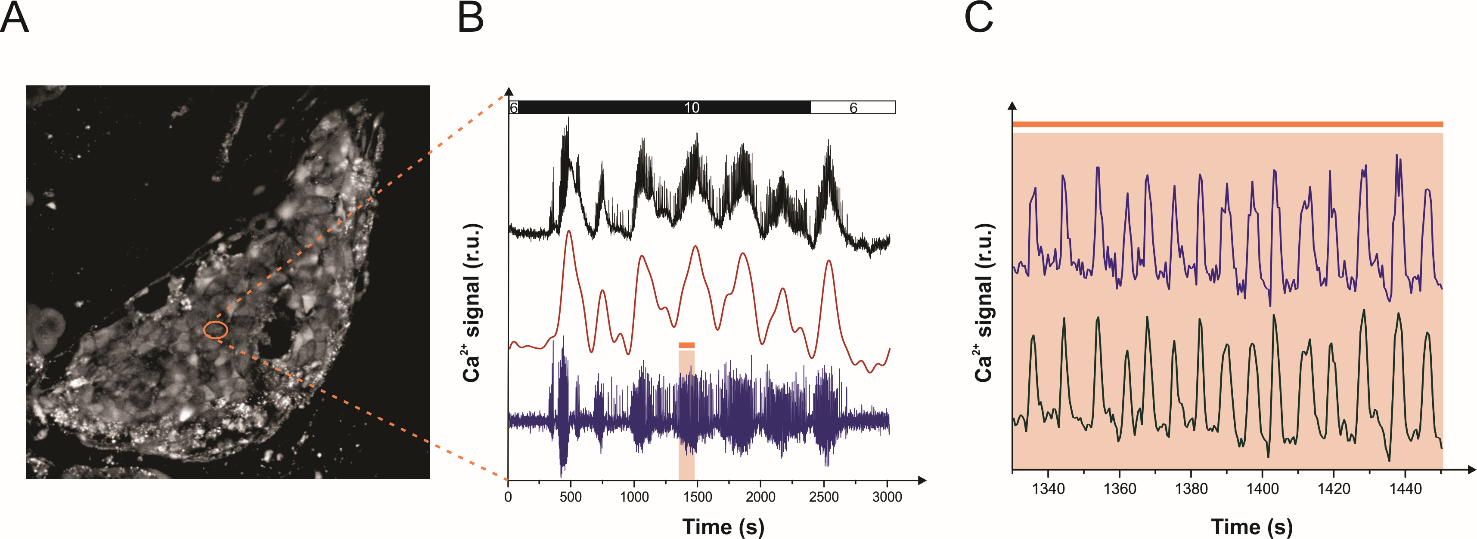


**Supplementary figure 1. Extraction of beta cell Ca^2+^ activity. (A)** The islet of Langerhans, observed through confocal microscopy. Beta cells, loaded with a calcium sensitive dye, are clearly distinguished. A representative beta cell is circumscribed by the orange circle. **(B)** The raw recorded trace, showing this cell response to stimulation, is depicted in black. Red and blue lines signify the extracted slow and fast Ca^2+^ dynamics, respectively. The subsequent analysis focused exclusively on the fast component of activity.  **(C)** A magnified view of the shaded segment in **(B)** displays the filtered fast activity in blue, while the signal after smoothing is presented in green. The smoothened signal underwent binarization for further analysis.


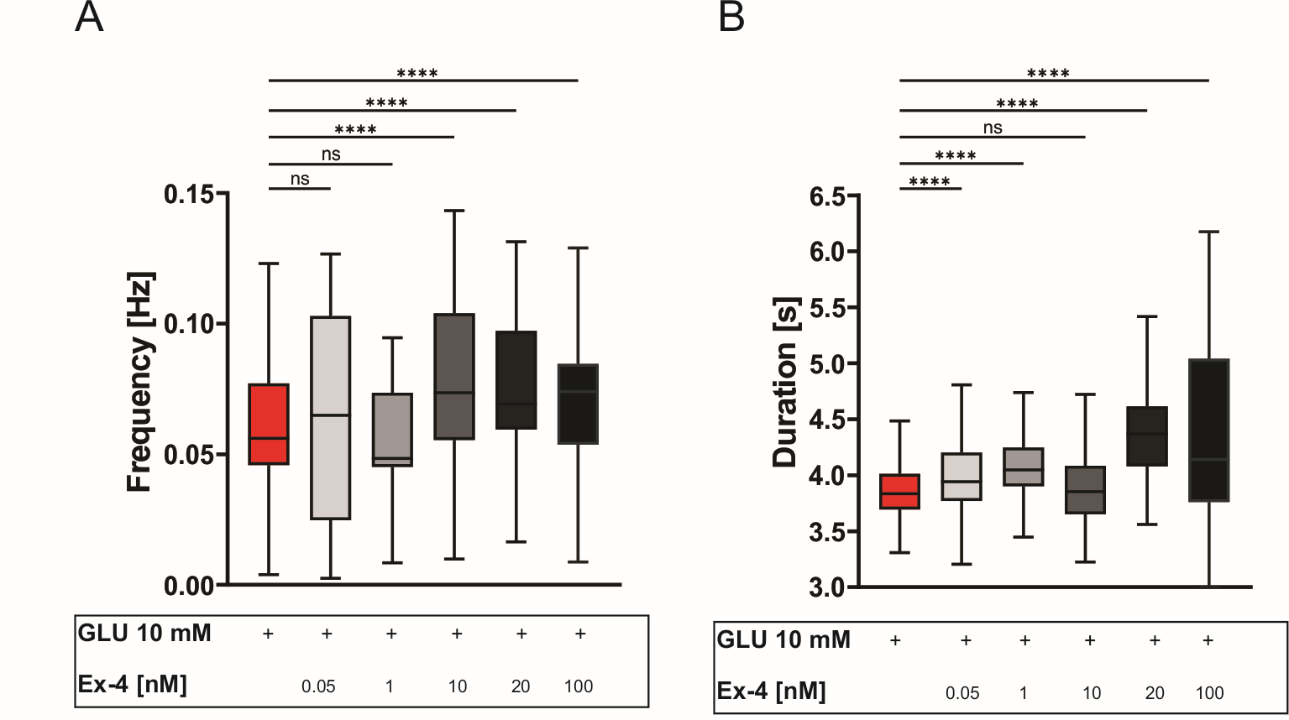


**Supplementary Figure 2. Plateau phase of the glucose response: the effect of Ex-4 costimulation. (A, B):** The effect of Ex-4 on frequency **(A)** and duration **(B)** of beta cell oscillations in interval 1. Median values for frequency (in Hz): 0.056 (10 mM glucose), 0.065 (0.05 nM), 0.048 (1 nM), 0.074 (10 nM), 0.069 (20 nM), 0.074 (100 nM) and duration (in seconds): 3.84 (10 mM glucose), 3.94 (0.05 nM), 4.05 (1 nM), 3.85 (10 nM), 4.37 (20 nM), 4.14 (100 nM). Cohen’s *d* values, reflecting the effect size are: 0.01 (0.05 nM), 0.23 (1 nM), 0.52 (10 nM), 0.48 (20 nM), 0.41 (100 nM) for beta cell frequency **(A)** and 0.4 (0.05 nM), 0.56 (1 nM), 0.08 (10 nM), 1.24 (20 nM), 0.66 (100 nM) for duration **(B)**. The data are pooled from 12 different pancreas preparations and the following number of cells/islets: 633/10 (10 mM glucose), 267/4 (0.05 nM), 288/4 (1 nM), 899/9 (10 nM), 191/3 (20 nM), 478/5 (100 nM). The following symbols indicate p-values: *p < 0.05, **p < 0.01, ***p < 0.001, ****p < 0.0001; ns, not significant. Effect size was interpreted as small (*d* = 0.2), medium (*d* = 0.5) or large (*d* = 0.8).
